# Allergic contact dermatitis to rubber accelerators in protective gloves: Problems, challenges, and solutions for occupational skin protection 

**DOI:** 10.5414/ALX02265E

**Published:** 2021-10-22

**Authors:** Andreas Hansen, Richard Brans, Flora Sonsmann

**Affiliations:** 1 *Department of Dermatology, Environmental Medicine and Health Theory, University of Osnabrück, and *; 2 *Institute for Interdisciplinary Dermatological Prevention and Rehabilitation (iDerm) at the University of Osnabrück, Germany*

**Keywords:** rubber accelerators, delayed-type allergy, thiurams, dithiocarbamates, thiazoles, guanidines, protective gloves, rubber, allergic contact dermatitis

## Abstract

Protective gloves are an elementary component of personal protective equipment in many occupations and are intended to protect the hands from various hazards (e.g., wetness, chemicals, mechanical forces, or thermal stress). This is particularly important when other occupational safety measures (e.g., technical-organizational measures) cannot be implemented or are insufficient. However, it is not uncommon for protective gloves themselves to become a problem, as some of their ingredients (e.g., rubber accelerators) can cause allergic reactions. Accelerators in rubber gloves include thiurams, dithiocarbamates, thiazoles, guanidines, and thioureas. If no alternative means of protection are available, this may even result in abandoning the profession. This article is about rubber accelerators, which are often contained in protective gloves made of different rubber materials (e.g., natural rubber (latex) and nitrile rubber) and may cause delayed-type allergies, as well as related challenges, problems, and solutions for occupational skin protection.

## Background 

In many occupations, wearing protective gloves is a fundamental component of occupational skin protection. Depending on the work activity, gloves are required as part of the personal protective equipment (PPE) to protect the hands against skin hazards, including wetness, chemicals, microbial contamination, soiling, mechanical or thermal stress. In the healthcare sector, they also protect patients from infectious diseases. The majority of liquid-tight glove models, such as disposable medical gloves and “chemical protective gloves”, are made of a rubber material, particularly natural rubber (latex), nitrile rubber, chloroprene rubber, or butyl rubber*. However, gloves that protect against mechanical impact, such as so-called assembly gloves, can also have a coating of natural or synthetic rubber. In the manufacturing of rubber products, accelerators are usually used to speed up the production process. As rubber accelerators can cause contact allergy (delayed-type hypersensitivity) and thus, allergic contact dermatitis, the use of protective gloves containing rubber accelerators could be problematic. This article discusses the challenges, problems and possible solutions that arise in selection of protective gloves for occupational purposes in the presence of contact allergy to rubber accelerators. 

*In general, the designations of the rubber materials are often shortened. For instance, nitrile rubber gloves are referred to as nitrile gloves. Following this practice, both forms are used synonymously in this article. 

## The vulcanization process in the production of elastomeric protective rubber gloves 

Vulcanizing agents, mainly sulfur but also sulfur-releasing substances such as dithiocarbamates, are used in the production of elastomeric rubber gloves. They serve to cross-link or polymerize the synthetic or natural rubber. To speed up the otherwise very slow process of vulcanization between the rubber and the vulcanizing agent, accelerators are added. These include thiurams, dithiocarbamates, thiazoles, guanidines, and thioureas [14]. Some accelerators are also used as vulcanizing agents. In the following, however, the term accelerator is used exclusively for these substances. In the process of vulcanization, the added accelerators may change, for example, through reduction and oxidation processes. Thiurams become dithiocarbamates and vice versa (redox pair); with the addition of thiazoles, completely new (intermediate) substances are formed with thiurams, such as sulfides (e.g., dimethylthiocarbamylbenzothiazole sulfide (DMTBS) and diethylthiocarbamylbenzothiazole sulfide (DETBS)), which themselves appear to cause sensitizations [3, 5, 12]. 

## Contact allergy to rubber accelerators 

Ingredients of protective gloves made of rubber materials can cause contact allergy (delayed-type hypersensitivity) and thus, allergic contact dermatitis. The most important contact allergens in gloves are rubber accelerators [14]. 

Table 1 provides an overview of rubber accelerators used in the manufacturing of protective gloves that cause contact allergy. 

In individuals with hand eczema using protective gloves made of rubber materials, patch testing should be performed to detect or exclude contact allergies to rubber accelerators (Table 2). The baseline series of the German Contact Dermatitis Research Group (DKG) already covers important accelerators as it contains the thiuram mix, the mercapto mix and 2-mercaptobenzothiazole. In addition, the DKG rubber series should be tested, which contains further accelerators in addition to the individual substances of the thiuram and mercapto mix. The additional testing of the single allergens of the mixes is important, because false-positive reactions towards the mixes occur and also reactions towards the single substances without reaction towards the mixes are observed [2, 8, 21]. It has been reported that ~ 20% of sensitizations to thiurams are not detected if only the thiuram mix is tested and not the individual substances [9]. In addition, patch testing of the unused patient’s own protective gloves is recommended, since not all glove allergens are available as commercial and approved test substances. As a rule, the inside and outside should be tested, as they may contain different ingredients. Pieces of the protective gloves can be tested “as is”, moistened with water under a tape (semi-open). Alternatively, testing can be performed after extraction in an ultrasonic bath [1]. In case of a positive reaction to the glove and missing reactions to the commercial test substances, a detailed investigation is required with an inquiry to the manufacturer about the glove ingredients and, if necessary, chemical analysis of the glove to identify the causative allergen. 

Contact allergy to accelerators is more frequent in occupational than in non-occupational dermatitis (adjusted prevalence risk: thiuram mix: 4.23, mercapto mix: 2.46, 2-mercaptobenzothiazole: 2.91) [16]. This is predominantly due to occupational use of liquid-tight protective gloves. Accordingly, contact allergy to accelerators is particularly common in occupational groups with long periods of wearing such protective gloves, including health care, food processing, or cleaning occupations [20]. Positive patch test reactions to thiurams are most frequent, although thiurams are hardly used any more in the production of protective gloves due to high sensitization rates [9, 18]. Sensitizations to dithiocarbamates, which are now predominantly used in the manufacturing of protective gloves, are found much less frequently [9, 12]. For example, retrospective analyses of patch test data from the Information Network of Departments of Dermatology (IVDK) from 2007 to 2018, revealed that positive test reactions to the thiuram mix were detected in 2% of patients and reactions to zinc diethyldithiocarbamate in only ~ 0.5% [19]. It should be noted here that thiurams and dithiocarbamates are chemically related and represent a redox pair [12]. Dithiocarbamates oxidize (for example with the help of iron ions) to the corresponding thiurams. Thiurams are reduced (for example under the influence of glutathione) to the corresponding dithiocarbamate. Thus, the continuing high number of thiuram sensitizations may indicate [15, 19, 20] that contact allergy to the thiuram/dithiocarbamate redox pairs can be better detected by patch testing of thiurams than by testing of dithiocarbamates [12]. This is also supported by the fact that nearly all persons with a positive reaction to dithiocarbamates concomitantly react to thiurams, whereas only about one third react the other way round. Furthermore, patch test reactions to dithiocarbamates are almost exclusively found in individuals with strong patch test reactions to thiurams [2]. 

Guanidines, especially 1,3-diphenylguanidine (DPG), are used as moderately fast accelerators in the polymerization of, for example, (poly)chloroprene/isoprene and butyl gloves and have long been among the less relevant contact allergens [9]. Due to an increasing share of (sterile) medical disposable gloves made of chloroprene/polyisoprene, the sensitization rate has increased in certain medical fields [1]. This is illustrated by publications from Belgium [7] and Sweden [17] as well as a recent case series from Germany [11]. However, patch test reactions to DPG have to be interpreted with caution as it belongs to the so-called “problem allergens” due to its irritant properties, and accordingly false-positive test reactions must be considered [9]. 

Thiazoles are increasingly used in protective gloves. So far, however, contact allergies to thiazoles have been detected much less frequently than to thiurams/dithiocarbamates [6, 21]. Thioureas are slow accelerators and are rather rarely used in gloves. Accordingly, contact allergies to thioureas are also rare [21]. 

## Challenge and possible solutions for glove selection 

In case of proven contact allergy to one or more accelerators, strict avoidance of the relevant accelerator group(s) is required. Depending on the field of activity and the materials handled at work, this may result in various challenges. Possible solutions are outlined below. Table 3 provides an overview. 

### Strategies in case of contact allergy to thiurams/dithiocarbamates, guanidines, and thiazoles 

In this case, contact with all rubber accelerators should be avoided. This is also an option in those with contact allergy to only part of the entire spectrum of accelerators. 

**Protective gloves without elastomeric rubber components**

An effective way to avoid any accelerators is to use gloves that do not contain rubber components such as latex, nitrile, or chloroprene, but are made of plastics such as polyvinyl chloride (PVC), vinyl, leather, polyvinyl alcohol (PVA), laminated film (LLDPE) or have a polyurethane (PU) coating. 

Depending on the occupational activity, this can be implemented without major challenges. For example, for dry, lightly soiling activities, a partially PU-coated glove can be used instead of a partially nitrile-coated assembly glove. However, if there is contact with moist or oily working materials, PU-coated gloves are not an adequate choice due to the rapid penetration of the working materials. 

For dry activities requiring little tactility, gloves made of a leather/textile mixture may be suitable. However, these are only suitable for a few areas of activity. In the case of leather gloves, it should also be noted that they are not suitable for individuals with sensitization to chromium due to the predominant use of leather tanning with chromium salts. 

For activities involving contact with liquids, liquid-tight gloves (in accordance with DIN EN ISO 374) made of PVC are a possible option. For instance, an everyday application of these gloves is dishwashing with commercially available detergents. Depending on the chemicals used, PVC gloves could also be suitable in a professional context. Even though gloves made of rubber materials usually have a higher resistance to various chemicals, the resistance of PVC gloves to some, but not all, acids and alkalis is also good. Whether the protection against agents used is adequate should be checked on a case-by-case basis. For example, PVC gloves do not provide sufficient protection against solvents (e.g., ethanol, nitro thinner). In addition, solvents can dissolve out the plasticizers contained in PVC gloves. Due to a partly higher thickness and a lower flexibility compared to many gloves made of rubber materials (e.g., nitrile or chloroprene), PVC gloves often offer a lower tactility and cause hand fatigue more quickly. Therefore, when selecting gloves, not only the suitability of the material for the respective agents, but also practicality from the user’s point of view should be considered. 

**Figure 1.**
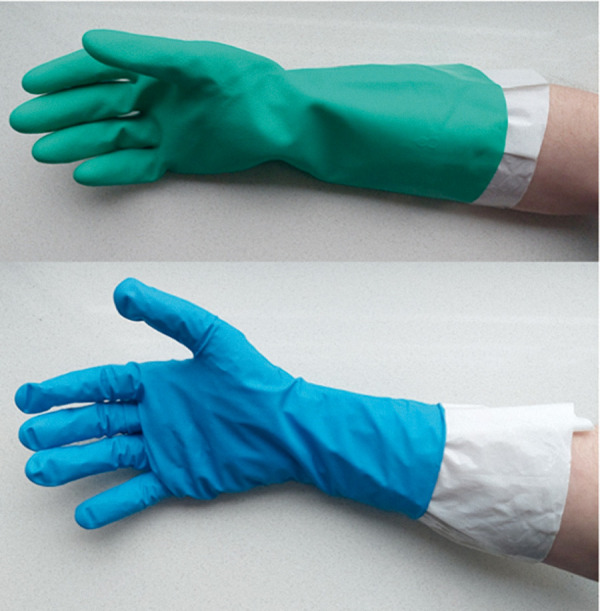
Linear low-density polyethylene (LLDPE) laminate film glove as an under-glove under a nitrile reusable glove and under a thick-walled nitrile disposable glove.

For adequate chemical protection, for example against nitro thinner or acetone, gloves containing accelerators made of natural or synthetic rubber are usually used (for example nitrile, chloroprene, butyl). However, protective gloves made of laminated film “linear low-density polyethylene” (LLDPE) can be an alternative. LLDPE gloves have very good resistance to a wide range of chemicals and are relatively inexpensive. One disadvantage of these glove models is that they are smooth 2D gloves with welded seams, which are uncomfortable for many users and also have poor wet and oil grip. In this case, the laminate gloves can be used together with cotton glove liners underneath and form-fitting wet or oil grip-resistant protective gloves on top (as outer layer) (Figure 1). 

**Accelerator-free rubber gloves**

In the field of partially coated assembly gloves, various manufacturers offer models with coatings free of rubber accelerators. For example, gloves with nitrile foam or aqua polymer coatings that meet different requirements (for example, handling dry, oily and slightly moist working materials) are available on the market. This applies to assembly gloves both with or without cut protection in accordance with DIN EN 388. In the case of nitrile disposable gloves, for example for healthcare, in accordance with DIN EN 455, or for food processing professions, models are available which, according to the manufacturer’s specifications, are produced free of rubber accelerators. However, the vast majority of these have a short cuff (approx. 24 cm). To the best of our knowledge, currently no thick-walled disposable gloves or reusable gloves made of one or more rubber materials without accelerators are available. 

**Polyethylene (PE) glove liners for protection against allergen exposure from protective gloves**

**Figure 2.**
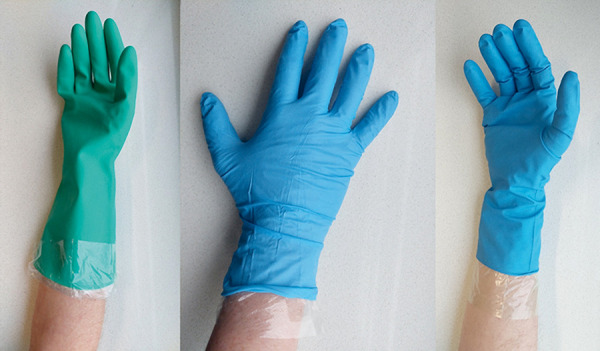
Polyethylene glove liner under a nitrile reusable glove, a nitrile medical disposable glove, and a nitrile thick-walled disposable glove.

**Figure 3. Figure3:**
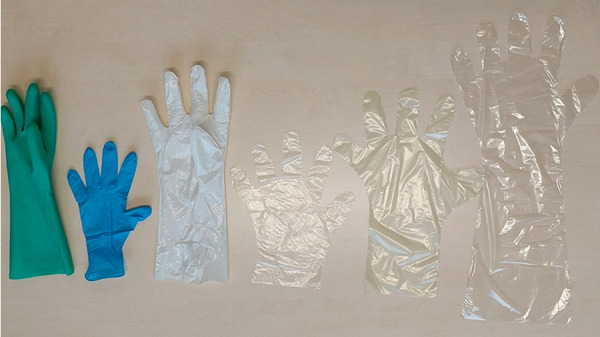
Nitrile reusable glove, nitrile disposable glove, linear low-density polyethylene (LLDPE) laminate film glove, and polyethylene gloves with different cuff lengths.

For many occupational activities, the use of protective gloves without a rubber accelerator is possible without difficulty. However, for adequate protection against various chemicals, thick-walled gloves made of rubber (e.g., nitrile, chloroprene) are often more suitable (e.g., in terms of chemical protection and practicality). With the exception of some disposable gloves, these contain one or more rubber accelerators. Particularly in cases of contact allergy to thiurams/dithiocarbamates, there is often no equivalent alternative available for protection against chemicals that is free of both groups of accelerators. One possible solution in these cases is to wear polyethylene (PE) gloves as glove liners underneath the accelerator-containing protective glove. In this way, skin contact with, for example, the nitrile reusable glove used for cleaning purposes or the thick-walled nitrile disposable glove used for prolonged surface disinfections can be avoided (Figure 2). The extent to which this basic option can be implemented should be decided individually case by case. It is necessary to take into account that the donning and doffing as well as the performance of the respective activities with the glove combinations must be feasible for the individual user. The working conditions in the company (e.g., work processes, possible time pressure, possibility of glove storage) should also be considered.[Fig Figure3]

### Strategies in case of contact allergy to thiurams/dithiocarbamates 

While thiurams are currently hardly ever used in glove production, dithiocarbamates are among the most frequently used vulcanization agents and rubber accelerators [12]. Accordingly, dithiocarbamates are now predominantly used in liquid-tight (according to DIN EN ISO 374) reusable gloves made of rubber materials as well as in thick-walled disposable gloves from various glove manufacturers. Thiurams may still be present in gloves made of butyl rubber, among others. If contact allergy to only one of the two groups of accelerators is detected, the other group should also be avoided because of the chemical relationship already described (redox pair). Due to the widespread use of dithiocarbamates, the choice of suitable gloves is significantly limited in cases of contact allergy to thiurams/dithiocarbamates. Only in a small proportion of glove models, a different accelerator (for example thiazoles) is used instead of thiurams/dithiocarbamates. Particularly in the handling of solvent-containing agents, the use of rubber-free gloves made of a “linear low-density polyethylene” (LLDPE, laminate) may be a possible alternative – depending on the duration of use and the field of activity (Figure 1). 

### Strategies in case of contact allergy to guanidines 

Guanidines, especially 1,3-diphenylguanidine (DPG), are used for example in (poly)chloroprene/isoprene and butyl gloves. Recently, increased cases of contact allergy to DPG have been reported in connection with (sterile) disposable medical gloves made of chloroprene/polyisoprene [7, 11, 17]. An experimental study showed that alcohol-based hand disinfectants, which are applied prior to the donning of sterile protective gloves, increase the release of DPG from the glove material [10]. This suggests that sterile gloves containing DPG should not be used for primary prevention reasons. There are alternative rubber gloves with other accelerators and also accelerator-free gloves, for example made of nitrile rubber and neoprene. 

### Strategies in case of contact allergy to thiazoles 

Thiazoles have been increasingly used in glove production in recent years [14]. If contact allergy is detected, protective gloves free of thiazoles must be used. This is usually less of a problem in the case of isolated sensitization to thiazoles, as a number of different models without thiazoles are available. 

### Strategies in case of contact allergy to thioureas 

Thioureas have so far been used rather rarely, for example in protective gloves made of neoprene/polyisoprene/chloroprene. There are many thiourea-free products available, so that the selection of suitable alternatives is generally not problematic. 

## Discussion of solutions for different professions 

### Health care, nursing, and geriatric care workers 

Medical disposable gloves (according to DIN EN 455) are required for typical nursing activities (e.g., basic nursing and wound care). In case of sensitization to one or more rubber accelerators, disposable vinyl gloves may be used as an alternative. However, due to the material properties (e.g., low extensibility, higher risk of microperforations), disposable vinyl gloves do not appear to be an equal alternative to disposable nitrile or latex gloves in these occupations. Recent developments indicate that a material mixture of vinyl and nitrile can do without accelerators. Gloves made of this material may be an alternative in these professions. The number of disposable nitrile glove models which are produced without rubber accelerators according to the manufacturer’s specifications has increased significantly in recent years. However, there are only a few models with an extended cuff (~ 28 – 30 cm). These long-cuff gloves can be useful, for example, for basic nursing activities in order to avoid the risk of water leaking into the glove. Various models (sterile and accelerator-free) are also available on the market for sterile nursing activities (e.g., when placing urinary bladder catheters) or assisting in the operation theatre. A challenge for the selection of suitable gloves can be surface disinfections. For very short surface disinfections, disposable nitrile gloves usually provide sufficient protection. However, as permeation times vary greatly depending on the agent and concentration, no general recommendation can be given for the use of (accelerator-free) disposable medical nitrile gloves for surface disinfections. In individual cases, it is therefore necessary to ask the glove manufacturer about the permeation time of the agent used. For longer lasting wipe disinfections, thick-walled disposable gloves are generally suitable, which usually provide longer lasting protection against surface disinfectants due to their higher thickness compared to medical disposable gloves. However, currently, corresponding models which are free of accelerators are not available. If it is therefore not possible to avoid disposable gloves containing rubber accelerators, it is advisable to use PE glove liners underneath to prevent direct skin contact with the disposable protective glove containing accelerators (Figure 2). Although this option may be practicable, it should always be considered on an individual basis taking into account the individual background of the user and the possibilities of implementation. 

### Employees in metalworking professions (e.g., lathe operators, drillers, tool makers) 

In metalworking professions, there are sometimes widely differing requirements for protective gloves so that the use of different models is often necessary. It must also be taken into account that, particularly in this occupational group, the wearing of protective gloves is not permitted for all activities for reasons of occupational health and safety. For example, employees who work wholly or partly on open rotating machines (e.g., conventional lathes) are not permitted to wear protective gloves because of the potential risk of them being trapped and drawn in. Whether gloves can and may be worn depends on the potential exposure to skin hazards. For dry to slightly oily or greasy activities with low mechanical requirements, partially coated assembly gloves with different coating thickness and types (e.g., nitrile foam for dry activities or aqua polymer for slightly oily activities) are required. Accelerator-free models that meet the requirements for these activities are available. This also applies to activities where additional protection against sharp-edged parts (for example sheet metal) is required. A challenge arises when liquid-tight or chemical-resistant gloves are required. For those who, for example, have increased contact with metalworking fluids when removing workpieces from the machine, it is often necessary to use protective gloves in accordance with DIN EN ISO 374. In order to maintain the grip, an additional grip coating, which is available on various glove models, can be useful. In case of contact allergy to thiurams/dithiocarbamates, the use of gloves made of PVC should be considered. With regard to chemical resistance, however, PVC gloves – as already mentioned – are often not equivalent to gloves made of rubber. Due to their material composition, particular thicker-walled PVC gloves with an additional grip coating are less flexible and therefore, require more force to be applied and consequently lead to hand fatigue more quickly. Liquid-tight rubber gloves contain usually at least one dithiocarbamate, so that they cannot be used in case of contact allergy to thiurams/dithiocarbamates. Only a few thick-walled gloves made of synthetic rubber material contain neither thiurams nor dithiocarbamates. In these cases, other rubber accelerators are often used (for example thiazoles or DPG). Provided there is no sensitization to other accelerators, these models may be an alternative. However, these glove models are not completely equivalent due to their material composition, chemical protection spectrum, and grip strength, among other things. Thus, it must be discussed in each individual case whether the use of these gloves is possible and expedient. An often more practicable application option here is the use of glove liners made of PE, in order to avoid skin contact with the accelerator-containing glove model. 

## Possibilities of obtaining information – Identification of rubber accelerators in gloves 

Information on rubber accelerators used in gloves can be found in some cases in technical data sheets. It should be noted that information is not provided in the same standardized way by all manufacturers. For example, data sheets with specific information on the accelerators used (e.g., zinc diethyl dithiocarbamate) can be found alongside data sheets from which it is clear that a certain group of vulcanization accelerators (e.g., dithiocarbamates) is used, without any further description of the individual substance. Some data sheets provide information on which rubber accelerators are not used (e.g., “free of thiurams”). Occasionally, it is also stated that rubber accelerators not listed are not used. Gloves made of rubber materials that are produced without accelerators are usually marketed as accelerator-free by the manufacturers. Claims such as “suitable for allergic subjects” and “hypoallergenic”, on the other hand, should be interpreted with caution, as they usually advertise the absence of latex proteins. Rubber accelerators may well be present in protective gloves labelled in this way. Particularly if the information does not seem plausible, a (supplementary) inquiry with the manufacturer may be helpful. Inquiries to the manufacturer are unavoidable if data sheets are not available, no information is given on the accelerators used, or contradictory information is provided. Inquiries to glove manufacturers should therefore be as specific as possible. 

Further assistance in identifying accelerators in protective gloves may be found, for example, in the “Allergen list by manufacturer” published by the German Social Accident Insurance Institution for the building trade (Berufsgenossenschaft der Bauwirtschaft) (available at: https://www-p2.bgbau.de/themen/sicherheit-und-gesundheit/gefahrstoffe/gisbau/allergene-in-schutzhandschuhen/allergenliste-nach-hersteller/; last accessed: July 19, 2021) or the brochure “Achtung Allergiegefahr BGI/GUV-I 8584”, published by the German Social Accident Insurance (Deutsche Gesetzliche Unfallversicherung: DGUV) (available at: https://www.gesundheitsdienstportal.de/files/GUV-I-8584-Allergiegefahr-durch-Latex-.pdf; last accessed: July 19, 2021). However, some of the lists are no longer up to date or distinguish only roughly according to allergen groups and not according to individual allergens; in addition, guanidines are not listed as potential glove allergens. 

## Conclusion and outlook 

In individuals with eczema of hands and possibly forearms who use protective gloves made of or with rubber at work as protection against skin hazards, allergic contact dermatitis caused by rubber accelerators should be confirmed or ruled out by patch testing. In addition to the DKG baseline and rubber series, the patient’s own protective gloves should be tested. If a contact allergy to one or more groups of rubber accelerators is present, this may result in considerable restrictions in selection of appropriate protective gloves. However, there are also different solutions, which must be considered individually, particularly with regard to practicality and usability at work. In many cases, suitable alternatives are available to keep the skin healthy and enable continuance of the profession. 

## Funding 

None. 

## Conflict of interest 

The authors declare no conflict of interest. 

**Table 1.** Overview of relevant groups of rubber accelerators and their individual allergens. Based on [1, 14, 22].

Group	Single allergens
Thiurams	Tetramethylthiuram disulfide
	Tetramethylthiuram monosulfide
	Tetraethylthiuram disulfide
	Dipentamethylene thiuram disulfide
Dithiocarbamates	Zinc diethyldithiocarbamate
	Zinc dibutyldithiocarbamate
	Zinc dimethyldithiocarbamate
	Zinc dipentamethylenedithiocarbamate
	Zinc dibenzyldithiocarbamate
	Zinc diisononyldithiocarbamate
Thiazoles	2-Mercaptobenzothiazole
	N-cyclohexyl-2-benzothiazyl sulfenamide
	2,2‘-Dibenzothiazyl disulfide
	2-(4-Morpholinyl mercapto)benzothiazole
Thioureas	Diphenylthiourea
	Dibutylthiourea
	Diethylthiourea
	Thiourea
Guanidines	1,3-diphenylguanidine
	Triphenylguanidine

**Table 2.** Vulcanization accelerators in the baseline and rubber series of the German Contact Dermatitis Research Group (DKG).

DKG baseline series		
Test substance	Concentration	Vehicle
Thiuram mix	1%	Petrolatum
Mercapto mix	1%	Petrolatum
2-Mercaptobenzothiazole	2%	Petrolatum
DKG rubber series		
Test substance	Concentration	Vehicle
Individual substances of the Thiuram mix: – Tetramethylthiuram disulfide – Tetramethylthiuram monosulfide – Tetraethylthiuram disulfide (disulfiram) – Dipentamethylene thiuram disulfide	0.25% 0.25% 0.25% 0.25%	Petrolatum Petrolatum Petrolatum Petrolatum
Single substances of the Mercapto mix – N-cyclohexyl-2-benzothiazyl sulfenamide – 2,2’-Dibenzothiazyl disulfide (MBTS) – 2-(4-Morpholinyl mercapto)benzothiazole	1% 1% 0.5%	Petrolatum Petrolatum Petrolatum
Zinc dibutyldithiocarbamate	1%	Petrolatum
Zinc dibenzyldithiocarbamate	1%	Petrolatum
Zinc diethyldithiocarbamate	1%	Petrolatum
1,3-diphenylguanidine	1%	Petrolatum
Diphenylthiourea	1%	Petrolatum
Dibutylthiourea	1%	Petrolatum

**Table 3.** Challenges and potential solutions in recommending protective gloves for delayed-type hypersensitivity to one or more vulcanization accelerators.

**Delayed-type hypersensitivity**	**Glove material as a potential source**	**Alternative recommendation options**	**Commentary, availability, usability and deployment limitations**
a. Thiurams/dithiocarbamates, guanidines, and thiazoles	Synthetic and natural rubber products: Nitrile, latex, butyl, viton, chloroprene/polyisoprene	Gloves made of other materials, for example polyvinyl chloride, vinyl, laminate, polyethylene, polyvinyl alcohol, assembly gloves with polyurethane coating (etc.)	In the case of reusable gloves (with the exception of the laminate glove), attention should be paid to possible limited chemical protection
			Liquid-tight plastic gloves are generally less flexible, thicker-walled and therefore less sensitive
			Vinyl disposable gloves often have poorer chemical resistance (e.g. in relation to hairdressing chemicals) and show lower resistance to viruses due to more frequent microperforations compared to disposable gloves made of rubber materials [13]
			The plasticizers contained in vinyl gloves may dissolve out in contact with fatty foods and pass into food with health concerns; therefore, vinyl gloves are of limited use for food processing [4]
		Designated accelerator-free protective rubber gloves	Various glove manufacturers offer short-cuff accelerator-free, non-sterile nitrile disposable gloves; long-cuff accelerator-free nitrile disposable gloves are occasionally available
			Various glove manufacturers offer accelerator-free sterile glove models (for example, made of neoprene)
			Thick-walled disposable gloves without accelerators (e.g., nitrile disposable gloves with a layer thickness of approx. 0.2 mm) are currently not available according to our information
			Thick-walled models known as liquid-tight reusable gloves made of accelerator-free rubber are not yet available.
			Assembly gloves with accelerator-free rubber coating are available
		Glove liners made of polyethylene for protection against glove allergens of the actual protective glove	Feasibility depends on the individual case
			Good practicability generally with relatively infrequent glove changes or if the polyethylene glove liner can be taken off and put on again together with the protective glove
			Mostly poor practicality when protective gloves need to be changed frequently, there is little time to put on and take off protective equipment and a high level of sensitivity is required
b. Thiurams/dithiocarbamates	Synthetic and natural rubber products; dithiocarbamates (common): nitrile, latex, butyl, viton, chloroprene/polyisoprene Thiurams (rare): for example in butyl	See alternatives under a.	In case of contact allergy to either substance group, the other group should also be avoided
		Protective gloves made of/with rubber without thiurams/dithiocarbamates	Sterile medical protective gloves (without thiurams and dithiocarbamates) in which guanidines are used as accelerators are available; from a primary prevention perspective, however, the avoidance of sterile gloves containing 1,3-diphenylguanidine appears to be sensible
			Possibly severe restrictions for those affected (see a.)
c. Guanidines	Mainly synthetic rubber products made from chloroprene/polyisoprene	See alternatives under a.	A wide range of protective gloves without guanidine exists
		Alternative protective gloves made of/with rubber without guanidine	
d. Thiazoles	Synthetic and natural rubber products: Nitrile, Latex, Butyl, Viton, Chloroprene/Polyisoprene	See alternatives under a.	Various protective gloves made of rubber without thiazoles are available
		Alternative protective gloves made of/with rubber without thiazoles	Restrictions for affected persons, if applicable (see a.)
e. Thioureas	Protective gloves made of neoprene/polyisoprene/chloroprene	See alternatives under a.	A large number of protective gloves made of rubber without thiourea are available
		Alternative protective gloves made of/with rubber without thiourea	Hardly any restrictions for those affected (see a.)
